# Accuracy validation of a wearable IMU-based gait analysis in healthy female

**DOI:** 10.1186/s13102-023-00792-3

**Published:** 2024-01-02

**Authors:** Yi He, Yuxia Chen, Li Tang, Jing Chen, Jing Tang, Xiaoxuan Yang, Songchuan Su, Chen Zhao, Nong Xiao

**Affiliations:** 1 Department of Rehabilitation Medicine, The First Affiliated Hospital of Chongqing Medical University, No.1 Youyi Road, Yuzhong District, Chongqing, 400016 China; 2 Department of Rehabilitation, Children’s Hospital of Chongqing Medical University, National Clinical Research Center for Child Health and Disorders, Ministry of Education Key Laboratory of Child Development and Disorders, Chongqing Key Laboratory of Pediatrics, No. 136 Zhongshan 2nd Road, Yuzhong District, Chongqing, 400016 China; 3 Department of Orthopedic Surgery, The First Affiliated Hospital of Chongqing Medical University, No.1 Youyi Road, Yuzhong District, Chongqing, 400016 China; 4 Shanqi (Chongqing) Smart Medical Technology Co., Ltd., Chongqing, China; 5 Chongqing Orthopedics Hospital of Traditional Chinese Medicine, Chongqing, China

**Keywords:** Gait analysis, Wearable inertial measurement unit, Optical motion capture

## Abstract

**Objective:**

The aim of this study was to assess the accuracy and test-retest reliability of a wearable inertial measurement unit (IMU) system for gait analysis in healthy female compared to a gold-standard optoelectronic motion capture (OMC) system.

**Methods:**

In our study, we collected data from 5 healthy young females. Participants were attached with markers from both the OMC system and the IMU system simultaneously. Data was collected when participants walked on a 7 m walking path. Each participant performed 50 repetitions of walking on the path. To ensure the collection of complete gait cycle data, a gait cycle was considered valid only if the participant passed through the center of the walking path at the same time that the OMC system detected a valid marker signal. As a result, 5 gait cycles that met the standards of the OMC system were included in the final analysis. The stride length, cadence, velocity, stance phase and swing phase of the spatio-temporal parameters were included in the analysis. A generalized linear mixture model was used to assess the repeatability of the two systems. The Wilcoxon rank-sum test for continuous variables was used to compare the mean differences between the two systems. For evaluating the reliability of the IMU system, we calculated the Intra-class Correlation Coefficient (ICC). Additionally, Bland-Altman plots were used to compare the levels of agreement between the two systems.

**Results:**

The measurements of Spatio-temporal parameters, including the stance phase (*P* = 0.78, 0.13, L-R), swing phase (*P* = 0.78, 0.13, L-R), velocity (*P* = 0.14, 0.13, L-R), cadence (*P* = 0.53, 0.22, L-R), stride length (*P* = 0.05, 0.19, L-R), by the IMU system and OMC system were similar. Which suggested that IMU and OMC systems could be used interchangeably for gait measurements. The intra-rater reliability showed an excellent correlation for the stance phase, swing phase, velocity and cadence (Intraclass Correlation Coefficient, ICC > 0.9) for both systems. However, the correlation of stride length was poor (ICC = 0.36, *P* = 0.34, L) to medium (ICC = 0.56, *P* = 0.22, R). Additionally, the measurements of IMU systems were repeatable.

**Conclusions:**

The results of IMU system and OMC system shown good repeatability. Wearable IMU system could analyze gait data accurately. In particular, the measurement of stance phase, swing phase, velocity and cadence showed excellent reliability. IMU system provided an alternative measurement to OMC for gait analysis. However, the measurement of stride length by IMU needs further consideration.

## Introduction

Gait analysis is a method used to evaluate human movement, with the aiming of assessing individuals’ walking patterns, postures, balance, and walking ability. By analyzing gait, information about individuals’ physiological status, motion capability, and potential health issues can be obtained [1–3]. Gait analysis is widely applied in various fields, including: (1) Assessing the walking ability of patients with neurological disorders [4–6], musculoskeletal diseases [7, 8], and geriatric dementia [9], (2) Developing personalized rehabilitation plans and assessing the progress of rehabilitation [10, 11], (3) Assisting athletes in improving walking techniques and postures to enhance their sports performance [12]. There are several technologies available for gait analysis, such as motion capture systems, pressure mats, and accelerometers. Motion capture systems play a crucial role in this analysis as they track human movements using multiple sensors and cameras, converting them into digital data [13].

Biomedical engineering research has proposed a gait analysis method using wearable sensors based on inertial measurement units (IMUs). Inertial sensors are the most common type of wearable sensor used for gait analysis [14–16]. The advantages of IMU systems include their smaller size and lower cost compared to Optical motion capture (OMC) systems, which has led to increased utilization in clinical diagnosis [17–20]. Previous studies have verified the feasibility of IMU. Park evaluated the validity of IMUs using statistical parametric mapping (SPM) for gait analysis, calculating and comparing lower-extremity joint angles of the hip, knee, and ankle measured by both IMUs and motion-capture systems (Mocap). Results suggest that IMU-based data can be used confidently during the stance phase [21]. A meta-analysis determined the concurrent validity and test-retest reliability of IMUs for measuring biomechanical gait outcomes during level walking in healthy adults. This study concluded that step and stride times measured by IMUs showed excellent validity and reliability [22]. Berber validated the within-day reliability of an IMU system for measuring lower limb gait kinematics and temporal-spatial parameters (TSP) in people with and without HIV, and concluded that IMU-based gait analysis is valid and reliable when applied to individuals with HIV. However, there are fewer studies comparing IMUs with Optical motion capture (OMC) systems. OMC systems are currently considered the gold standard for motion signal capture [22, 23], widely used in human motion capturing and gait analysis [24–26]. However, OMCs have several limitations in clinical applications. They are relatively expensive, require complicated operation, and are too large to be used outdoors [27]. Therefore, wearable systems that are easy to move are preferred [22, 28].

In order to increase the universal use of the IMU system, it is very necessary to analyze the accuracy of the IMU system by comparing the IMU system and the OMC system. Previous studies mainly focused on patients with gait impairment. It may be more accurate and stable to evaluate IMU and OMC with gait analysis data from healthy adult. Therefore, the aim of this study was to evaluate the accuracy and retest reliability of the wearable IMU system compared to the gold standard OMC system, by performing gait analysis on healthy subjects.

## Method

### Design

This study was conducted as a validation study. Gait analysis was performed on all enrolled participants using both IMU and OMC systems simultaneously. Two trained technicians performed all measurements. All participants provided informed consent. The study protocol was evaluated and approved by the medical ethics committee, with the approval number 20,210,142.

### Participants

The study inclusion criteria were: [1] age between 18 and 30 years, [2] no history of neurological or musculoskeletal disease and [3] no medical problem associated with the balance and gait. All participants with informed written consent. Five healthy females meet inclusion criteria and included in the final analysis. The mean age of participants was 25.4 years (SD: 3.21), and the mean height and weight were 1.57 m (SD: 2.73) and 52.2 kg (SD: 5.89), respectively.

### Preparation procedure—IMU system setup

**Fig. 1:**
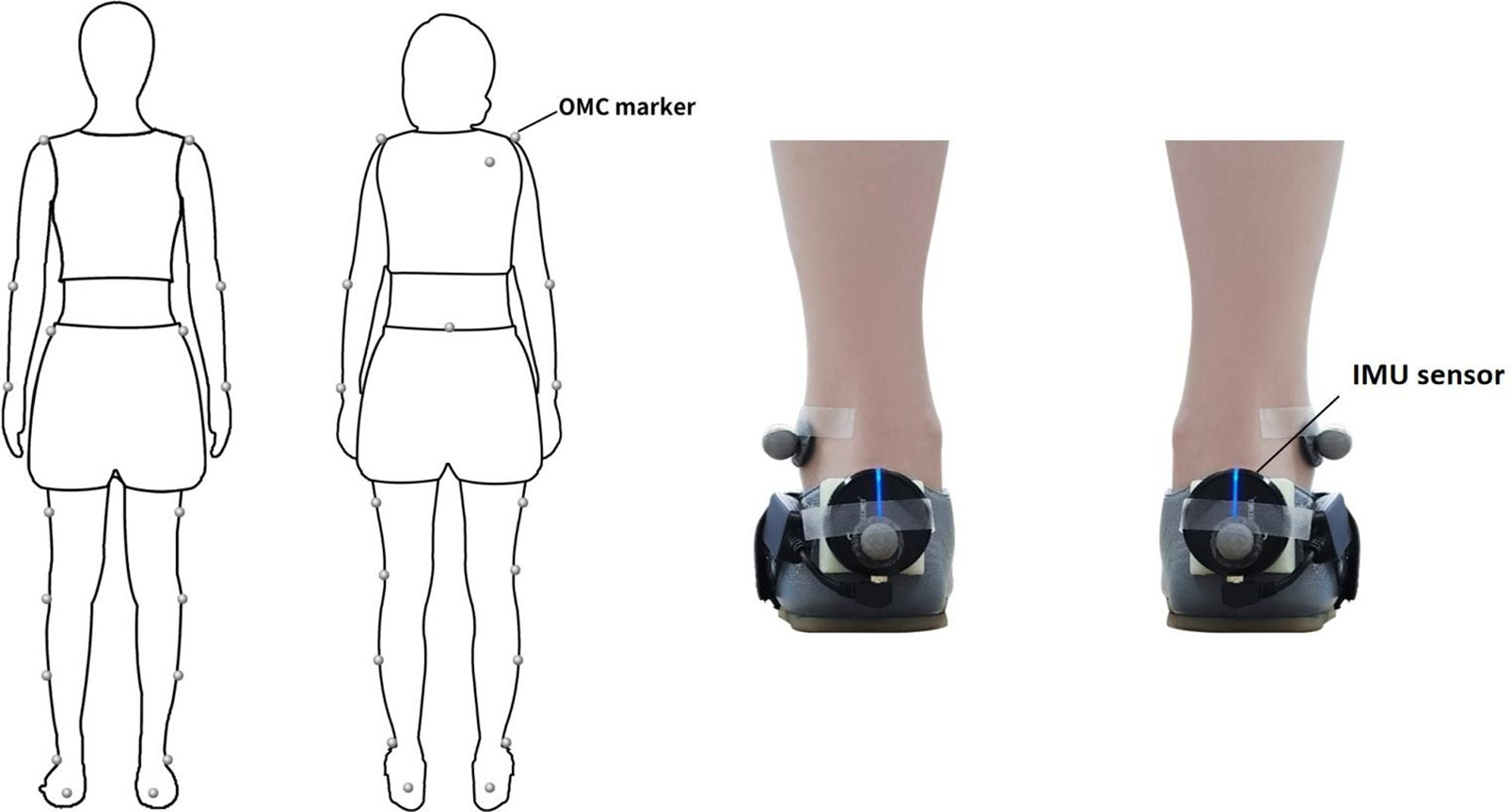
Sensor and marker positions in the inertial measurement unit (IMU) and optical motion capture (OMC) systems

Participants were equipped with IMU system. The IMU system includes both an IMU sensor and a plantar pressure sensor. In order to maintain the stability of stride length and stride velocity, the IMU sensor was attached to the heel positions and was positioned closer to the ground (Fig. 1). The IMU sensors were composed of a 3- axis accelerometer, a 3-axis gyrometer and a 3-axis magnetometer, respectively mounted on the heel. A plantar pressure sensor is connected to a specialized sole pressure insole to transmit signals of foot pressure. Foot Secret gait analysis equipment (Shanqi Wisdom Medical Technology Co. LTD, China) was used to measure Spatio-temporal parameters of gait.

### Preparation procedure—OMC system setup

The reflective markers tracked by an OMC system were used simultaneously to assess the accuracy of the IMU. In total, 22 markers were attached to the bilateral acromion, elbow, wrist, anterior superior iliac spine, middle femur, knee joint, middle tibia, external ankle, heel, toe and right scapular, lumbosacral during measurement (Fig. 1). The analysis was conducted using Qualisys systems, which utilized Qualisys Tracking Manager (QTM) motion capture technology to obtain 3D coordinates of reflective markers.

### Data extraction and processing

Participants had markers of the OMC system and the IMU system attached simultaneously. Data were collected when participants walked on a 7 m walking path. After a familiarization period, participants walked on the walking path at a comfortable speed. Each participant performed 50 repetitions of walking on a 7-meter walking path. The measurement was repeated 50 times to avoid the bias of participants (e.g., nervous or maladaptive) or technical problems (e.g., sensor unstable or fault). The IMU system recorded all the gait cycle data throughout the process.

The OMC system was fixed at the midpoint of the walking path. Only if a participant passed through the center of the walking path at the same time that OMC detected a valid marker signal with a gait cycle, could a complete gait cycle data be captured and collected. 5 gait cycles that meet OMC system standards will be included in the final analysis, the corresponding data of these 5 gait cycles were extracted for test-retest reliability analysis from IMU system. The stride length, cadence, velocity, stance phase and swing phase of the spatio-temporal parameters were included in the analysis.

### Statistical analysis

All statistical analyses were performed using SPSS 21.0. Bland–Altman plots were used to evaluate the same group of subjects by two different systems and intuitively reflect the agreement levels of gait analysis results obtained from the IMU and OMC systems [29, 30]. The mean differences between the two systems were compared using the Wilcoxon rank-sum test for continuous variables. ICC (Intra-class Correlation Coefficient) was used to quantify the reliability of IMU. The ICC value ranges between 0 and 1, where 0 represents no reliability and 1 represents complete reliability. A reliability coefficient below 0.4 indicates poor reliability, while a coefficient above 0.75 indicates good reliability. The generalized linear mixture model was used to analyze the repeatability of IMU and OMC system respectively. The level of significant difference was set at *P* < 0.05.

## Results

Each participant was measured five times using IMU and OMC systems, respectively, to verify the repeatability. The IMU system showed good repeatability using generalized linear mixture model for the stance phase (*P* = 0.75, 0.18, L-R), swing phase (*P* = 0.75, 0.51, L-R), velocity (*P* = 0.12, 0.41, L-R), cadence (*P* = 0.83, 0.64, L-R), stride length (*P* = 0.72, 0.81, L-R) (Table 1). OMC system also showed good repeatability using the generalized linear mixture model, in stance phase (*P* = 0.24, 0.56, L-R), swing phase (*P* = 0.24, 0.56, L-R), velocity (*P* = 0.78, 0.19, L-R), cadence (*P* = 0.45, 0.91, L-R), stride length (*P* = 0.65, 0.33, L-R) (Table 1).

**Table 1:** Repeatability of data acquisition for IMU system and OMC system

	Stance phase (%)		Swing phase (%)		Velocity (cm/s)		Cadence (steps/min)		Stride length (cm)	
	L	R	L	R	L	R	L	R	L	R
IMU	0.75	0.18	0.75	0.51	0.12	0.41	0.83	0.64	0.72	0.81
OMC	0.24	0.56	0.24	0.56	0.78	0.19	0.45	0.91	0.65	0.33

IMU: inertial measurement unit system; OMC: optical motion capture

The comparison of gait spatiotemporal parameters in the IMU and OMC systems were analyzed by Wilcoxon rank sum test, as shown in Table 2. The measurements of Spatio-temporal parameters, including the stance phase (*P* = 0.78, 0.13, L-R), swing phase (*P* = 0.78, 0.13, L-R), velocity (*P* = 0.14, 0.13, L-R), cadence (*P* = 0.53, 0.22, L-R), stride length (*P* = 0.05, 0.19, L-R), by the IMU system and OMC system were similar. Regarding the ICC of the IMU systems compared with the OMC system, ICC was used to evaluate the correlation of the IMU system relative to the OMC system as shown in Table 3. The intra-rater reliability showed an excellent correlation for the stance phase, swing phase, velocity and cadence (Intraclass Correlation Coefficient, ICC > 0.9) for both systems. However, the correlation of stride length was poor (ICC = 0.36, *P* = 0.34, L) to medium (ICC = 0.56, *P* = 0.22, R).

**Table 2:** The comparison of the gait spatiotemporal parameters between IMU and OMC systems by Wilcoxon rank-sum test

	stance phase(%)		swing phase(%)		Velocity(cm/s)		Cadence(steps/min)		stride(cm)	
	L	R	L	R	L	R	L	R	L	R
IMU	60.3	61.89	39.7	38.11	109.6	109.16	113.00	114.00	116.66	113.63
	(2.50)	(3.22)	(2.50)	(3.22)	(11.94)	(12.03)	(12.00)	(10.00)	(9.12)	(9.83)
OMC	60.00	62.50	40.00	37.50	112.44	112.68	116.13	116.13	117.89	114.95
	(5.16)	(4.13)	(5.16)	(4.13)	(12.46)	(15.62)	(13.79)	(17.03)	(4.02)	(5.37)
z	-0.28	-1.50	-0.28	-1.50	-1.48	-1.51	-0.62	-1.23	-1.99	-1.32
*p*	0.78	0.13	0.78	0.13	0.14	0.13	0.53	0.22	0.05	0.19

Values represent Median (Interquartile Range)

IMU: inertial measurement unit system; OMC: optical motion capture

**Table 3:** The ICC of IMU systems compared with OMC system

	stance phase (%)		swing phase (%)		Velocity(cm/s)		Cadence(steps/min)		stride length(cm)	
	L	R	L	R	L	R	L	R	L	R
ICC	0.98	0.97	0.98	0.97	0.95	0.94	0.98	0.96	0.36	0.56
*p*	<0.01	<0.01	<0.01	<0.01	<0.01	<0.01	<0.01	<0.01	0.34	0.22

IMU: inertial measurement unit system; OMC: optical motion capture

ICC: Intra-class Correlation Coefficient

**Fig. 2:**
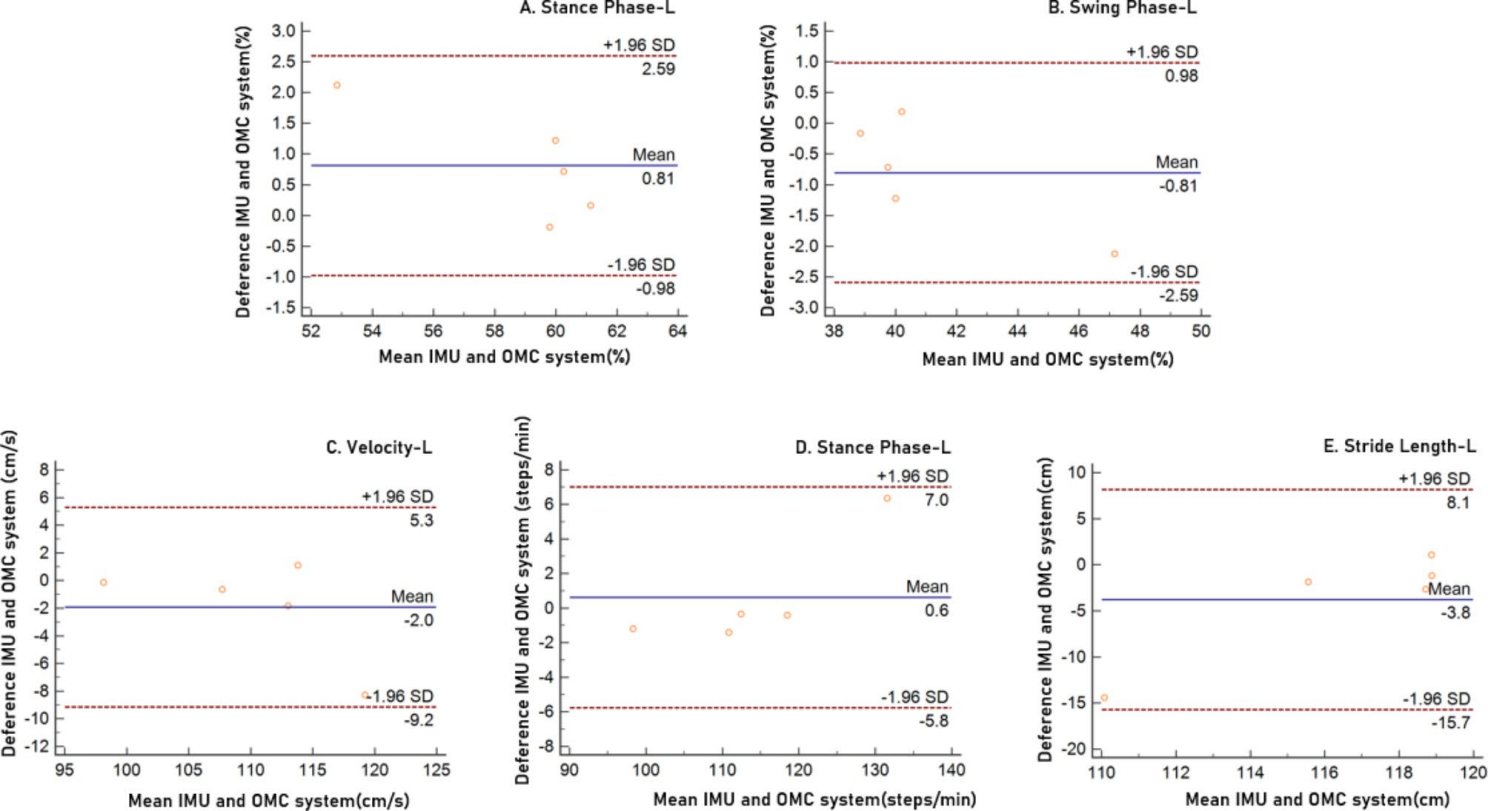
Bland–Altman plots comparing IMU system and OMC system results for (**A**) Stance Phase, (**B**) Swing Phase, (**C**) Velocity, (**D**) Cadence Step, and (**E**) Stride Length left. Bias (solid line) and limits of agreement are (dashed line) shown for each variable. The mean score is plotted on the x-axis, and the difference between the two devices is plotted on the y-axis (mean difference ± 1.96 SD)

The mean differences between the IMU and OMC systems for the stance phase, swing phase, velocity, cadence step and stride length left were 0.81, -0.81, -2.0, 0.6, and − 3.8, respectively. In the Bland–Altman plots, the limit of agreement for the stance phase, swing phase, velocity, cadence step and stride length left were 2.59 to -0.98, 0.98 to -2.59, 5.3 to -9.2, 7.0 to -5.8, and 8.1 to -15.7, respectively. All Spatio-temporal parameters were within a 95% limit of agreement from the means of differences between the IMU and OMC systems (Fig. 2).

**Fig. 3:**
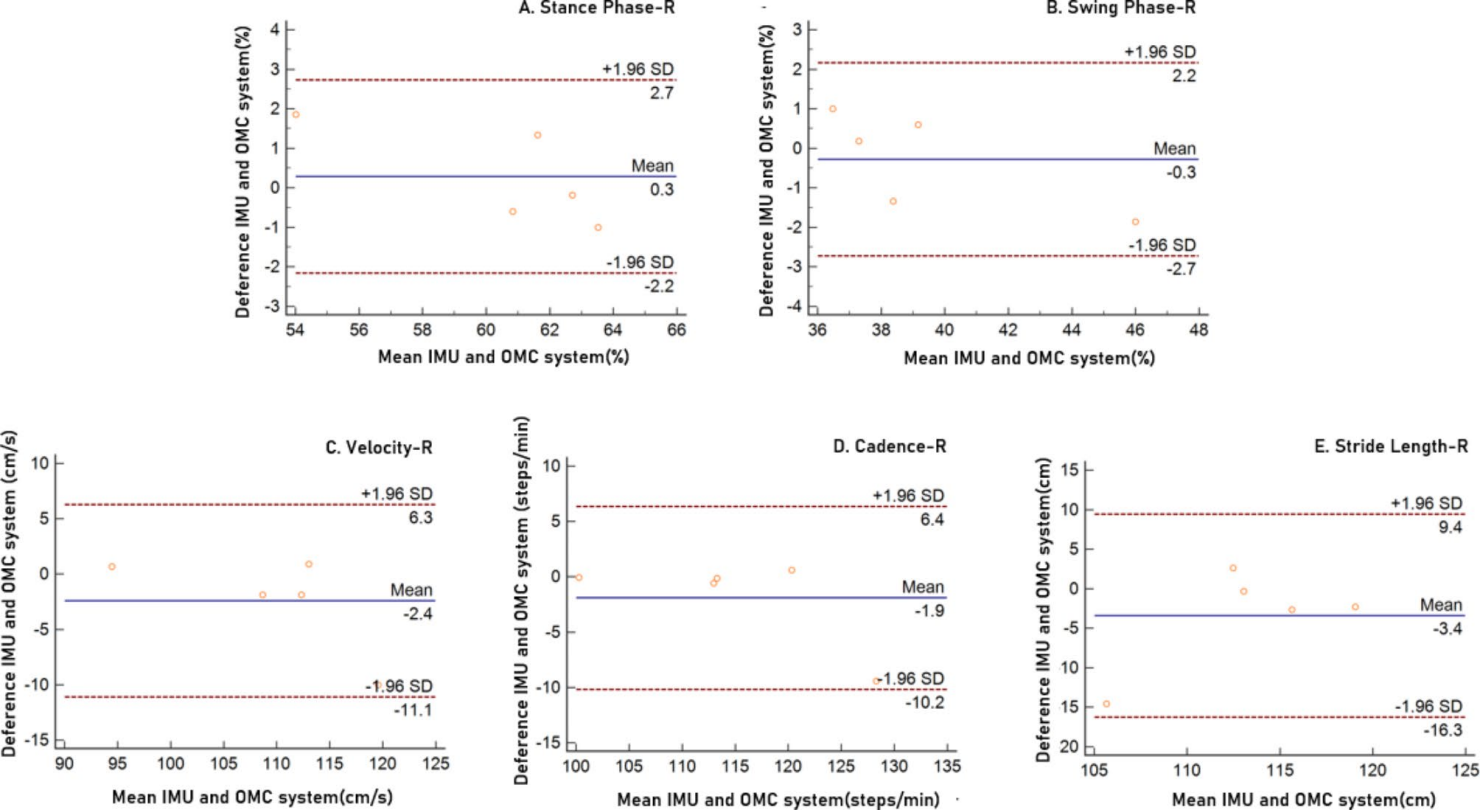
Bland–Altman plots comparing IMU system and OMC system results for (**A**) Stance Phase, (**B**) Swing Phase, (**C**) Velocity, (**D**) Cadence Step, and (**E**) Stride Length right. Bias (solid line) and limits of agreement are (dashed line) shown for each variable. The mean score is plotted on the x-axis, and the difference between the two devices is plotted on the y-axis (mean difference ± 1.96 SD)

The mean differences between the IMU and OMC systems for the stance phase, swing phase, velocity, cadence step and stride length right were 0.3, -0.3, -2.4, -1.9, and − 3.4, respectively. In the Bland–Altman plots, the limit of agreement for the stance phase, swing phase, velocity, cadence step and stride length right were 2.7 to -2.2, 2.2 to -2.7, 6.3 to -11.1, 6.4 to -10.2, and 9.4 to -16.3, respectively. All Spatio-temporal parameters were within a 95% limit of agreement from the means of differences between the IMU and OMC systems (Fig. 3).

The data acquisition efficiency during gait in the IMU system and OMC system iis shown in Table 4. For the IMU system, the average gait cycle in 20 min was 282, with an average time of 1.78 s for 1 gait cycle. For the OMC system, the average gait cycle in 20 min was 10, with an average time of 360 s for 1 gait cycle.

**Table 4:** Data acquisition efficiency during gait in the IMU system and OMC system

	Gait cycles acquired in 20 min (steps)		Time required for 1 gait cycle (sec)	
	IMU	OMC	IMU	OMC
A	334	10	1.86	360
B	248	10	1.54	360
C	266	10	1.80	360
D	280	10	2.04	360
E	282	10	1.71	360
Average	282	10	1.79	360

IMU: inertial measurement unit system; OMC: optical motion capture

## Discussion

In this study, we aimed to assess the accuracy and test-retest reliability of a wearable inertial measurement unit (IMU) system for gait analysis in healthy female, comparing to a gold-standard optoelectronic motion capture (OMC) system. The generalized linear mixture model demonstrated that both the IMU and OMC systems exhibited good repeatability.

Bland–Altman plots were utilized to compare the coincidence levels of the two systems [31]. The results indicated that all points were within a 95% limit of agreement (within the two lines of mean ± 1.96 SD). This indicates a high degree of consistency in the spatio-temporal parameters between the IMU and OMC systems, suggesting that the two systems are interchangeable [29, 32].

Furthermore, we conducted intraclass correlation coefficient (ICC) analysis for quantitative comparison and found that the measurement of IMU systems in the stance phase, swing phase, velocity, and cadence exhibited excellent reliability. However, it is important to note that the measurement of stride length by the IMU system may require further consideration. This parameter showed less reliability and may be influenced by factors such as individual differences in height and weight. Future research should focus on improving the accuracy and reliability of stride length measurements using IMU technology.

The findings of this study are consistent with previous studies that have validated or examined the repeatability of wearable IMU sensors for gait analysis. A meta-analysis, which included 82 articles and assessed the validity and reliability of IMUs across over 100 outcomes, found that the validity and reliability of step and stride times were excellent. Additionally, the validity and reliability of step and stride length, as well as swing and stance time, were rated as good to excellent. The results of this meta-analysis provided strong evidence for the excellent validity and reliability of IMUs for mean spatiotemporal parameters during walking [22].

Washabaugh conducted a study with 39 healthy subjects and concluded that the IMU system demonstrated accuracy and repeatability in measuring spatio-temporal gait parameters in healthy young adults [17]. It is worth noting that this study evaluated gait parameters using equipment and software specifically from APDM Opal IMUs and Mobility Lab system, and caution should be exercised when generalizing the results to IMU systems other than APDM.

Similarly, Yeo conducted a study in which they measured spatiotemporal and kinematic parameters during normal walking in young adults. The study aimed to assess the accuracy of an IMU system for gait analysis by comparing it with measurements obtained using an optical motion capture (OMC) system [33]. The results showed that the measurements of spatiotemporal and kinematic parameters of gait obtained from the IMU and OMC systems were similar. However, there is a difference between our study and the mentioned study in terms of the specific spatiotemporal parameters analyzed. We focused on parameters such as stance phase, swing phase, velocity, cadence step, and stride length, while they analyzed parameters such as stride time, stride length, cadence, and step length. Despite these differences, both studies arrived at similar conclusions.

A common conclusion from our study and previous research is that wearable IMU systems have the potential to efficiently provide gait measurements and enable accurate analysis. This suggests that IMU systems have significant potential for application in clinical gait analysis [15, 21, 34].

The above comparison of IMU and OMC is based on healthy adults because their gait data is stable. The same outcomes were found in patients after total hip arthroplasty [35]. The root mean squared errors in the joint kinematics from 0.24° to 1.25° in IMU system-based kinematic feature. The validity of the spatio-temporal gait parameters showed high accuracy. In our study, IMU system has a higher data extraction rate than other systems. As shown in Table 4, the number of effective gait cycles obtained by IMU is about 28 times that of OMC in the same time. The IMU sensor is a combination of accelerometer and gyroscope sensors. It is used to detect acceleration and angular velocity to indicate motion and intensity of motion. The advantage of IMU is that it can record gait data throughout the process, which makes up for the shortcomings of OMC.

## Limitations

The present study has several limitations that should be acknowledged. Firstly, the small sample size limits the generalizability of our findings. With a small sample, there may exist potential biases and the results may not be representative of the larger population. Therefore, we will strive to increase the sample size in future research, in order to better represent the target population and improve the reliability and stability of statistical analysis.

Secondly, the study participants were limited to female adults, which might restrict the generalizability of the results to other populations. To obtain more comprehensive and applicable results, we will introduce data collection from participants with diverse characteristics to enhance the heterogeneity of the sample in future studies. This will contribute to a more comprehensive understanding of the accuracy of the IMU system in gait data across different populations.

Thirdly, it is important to note that IMU systems are primarily designed to detect ankle movements, and whole body data cannot be accurately captured. As a result, the comparison of data across individuals with different heights and weights may introduce errors and inconsistencies. It is crucial to consider these limitations when interpreting and applying the findings of the study to diverse populations.

## Conclusions

The results of our study indicated that the IMU system and OMC system exhibited good repeatability. This suggests that the wearable IMU system is capable of accurately analyzing gait data. Specifically, the measurement of stance phase, swing phase, velocity, and cadence demonstrated excellent reliability. Therefore, the IMU system can serve as an alternative measurement tool to the OMC system for gait analysis. However, the measurement of stride length by the IMU system may require further consideration.

## Data Availability

The datasets used is available from the corresponding author on reasonable request.

## Abbreviations

IMU: Wearable inertial measurement unit
OMC: Optoelectronic motion capture
QTM: Qualisys Tracking Manager
ICC: Intra-class Correlation Coefficient
